# Reduced expression of autophagy markers correlates with high-risk human papillomavirus infection in human cervical squamous cell carcinoma

**DOI:** 10.3892/ol.2014.2417

**Published:** 2014-08-05

**Authors:** HUA-YI WANG, GUI-FANG YANG, YAN-HUA HUANG, QI-WEN HUANG, JUN GAO, XIAN-DA ZHAO, LI-MING HUANG, HONG-LEI CHEN

**Affiliations:** 1 Department of Pathology, Jingzhou Second People’s Hospital, Jingzhou, Hubei 434000, P.R. China; 2 Department of Pathology, Zhongnan Hospital of Wuhan University, Wuhan, Hubei 430071, P.R. China; 3 Department of Molecular Pathology, Wuhan Nano Tumor Diagnosis Engineering Research Center, Wuhan, Hubei 430075, P.R. China; 4 Department of Pathology, School of Basic Medical Science, Wuhan University, Wuhan, Hubei 430071, P.R. China; 5 Department of Oncology, Zhongnan Hospital of Wuhan University, Wuhan, Hubei 430071, P.R. China

**Keywords:** autophagy, cervical cancer, beclin-1, light chain 3B, human papilloma virus, quantum dots

## Abstract

Infection by an oncogenic human papillomavirus (HPV), in particular HPV16 and 18, is a high risk factor for developing cervical cancer; however, viral infection alone is not sufficient for cancer progression. Autophagy is hypothesized to be an important process during carcinogenesis. The aim of the present study was to investigate the association between autophagy and high-risk HPV (hrHPV) infection in human cervical squamous cell carcinomas (SCCs), and to analyze the clinical significance of this association. Quantum dot (QD)-based immunofluorescence histochemistry was used to detect the expression of autophagy markers, Beclin-1 and microtubule-associated proteins 1A/1B light chain 3B (LC3B) proteins, in 104 cases of cervical cancer (including 80 SCCs and 24 adenocarcinomas) and 20 normal cervical tissues. hrHPV (HPV16/18) infection was detected by QDs based fluorescence *in situ* hybridization in cervical cancers. The results revealed that the expression levels of Beclin-1 and LC3B were significantly lower in cervical cancer cells when compared with those of normal cervical squamous epithelial cells, and were found to negatively correlate with hrHPV infection. The expression levels of Beclin-1 and LC3B were not associated with age, tumor grade, tumor stage, tumor node metastasis stage or lymph node metastasis. However, a positive correlation was identified between Beclin-1 and LC3B protein expression. In addition, the absence of autophagy in combination with hrHPV infection may accelerate the progression of cervical SCC. In conclusion, decreased expression of Beclin-1 and LC3B may be important in cervical carcinogenesis. The hrHPV-host cell interaction may inhibit autophagy, which may aid virus duplication and infection, as well as cervical cancer development.

## Introduction

Cervical cancer is a leading cause of morbidity and mortality in females worldwide (1,2), and remains the most common type of gynecological malignant tumor in China (3). Infection by an oncogenic human papillomavirus (HPV), in particular high-risk HPV (hrHPV) types 16 and 18, is a risk factor for developing cervical cancer (4). HPV is a double-stranded DNA virus, which infects cutaneous and mucosal epithelial cells of the human body. HPV genomes consist of early, late, upstream regulatory and non-coding regions. These viruses usually clear in 70–90% of HPV infected individuals. Although HPV is the major causative agent of cervical cancer, the viral infection alone is not sufficient for cancer progression (5). Investigating the underlying cellular and molecular mechanisms in cervical carcinogenesis is a key aim of studies worldwide in the vaccine era.

Autophagy is a self-degradation mechanism, which is associated with tumor progression, including cervical cancer. Autophagy is a highly conserved intercellular process in all eukaryotes, from yeast to humans (6). Autophagy occurs as a result of certain stresses or inductions, including nutrient starvation, radiation or cytotoxic compounds. Subsequently, cytoplasm components or organelles are delivered to a double-membrane vesicle (autophagosome), which are then fused with lysosomes for protein degradation (7). To date, autophagy has been found to be involved in a variety of physiological and pathophysiological processes, including cell survival, cell death, tumor suppression and metastasis, antigen presentation, pathogen clearance, anti-aging and neurodegeneration (8–11).

Beclin-1 and microtubule-associated protein 1A/1B light chain 3 (LC3) genes exhibit a pivotal role in mammalian autophagy (12). Beclin-1, a mammalian orthologue of yeast Atg6, is involved in the signaling pathway which activates autophagy and in the initial step of autophagosome formation (13). LC3 comprises a soluble form, LC3A, and a lipidated form, termed LC3B. LC3B has been found to correlate with autophagy, and is recruited into autophagosomes. In addition, LC3B protein is widely used as a marker of autophagy in diverse cell types (14,15). Hence, in this study, Beclin-1 and LC3B expression in cervical cancer and precancerous lesions was evaluated. Targeted manipulation of complex autophagic signaling may present an innovative strategy for the identification of clinically relevant biomarkers in cervical cancer in the future.

We hypothesized that autophagy reduction may promote the development of cervical squamous cell carcinoma (SCC), particularly following hrHPV infection. Using the established quantum dot (QD)-based immunofluorescence histochemistry (IHC) technique, the expression of Beclin-1 and LC3B in cervical cancer and the correlation with clinicopathological parameters of cervical SCC was investigated. hrHPV infection was also detected using QD-based fluorescence *in situ* hybridization (FISH) to investigate the association between autophagy and hrHPV infection.

## Materials and methods

### Patients and tissue samples

A total of 124 formalin-fixed, paraffin-embedded (FFPE) cervical lesion samples, including 80 cases of cervical SCC and 24 cases of cervical adenocarcinoma, obtained from patients who underwent total hysterectomy due to complaints other than uterine cervical lesions, including uterine fibroids and endometriosis, and 20 cases of normal cervical tissues were used in this study. All study samples were obtained from patients between 2007 and 2010 who were treated at Zhongnan Hospital of Wuhan University (Wuhan, China). Two pathologists (Yang GF and Chen HL) reconfirmed the histopathological features of these samples. The histological diagnosis and grading of cervical cancers was established according to the International Federation of Gynecology and Obstetrics (FIGO) guidelines (Table I) (16). Approval for this study was obtained from the Ethics committee of Wuhan University. Written informed consent was obtained from the patient.

### Tissue microarray (TMA) construction

Hematoxylin and eosin-stained sections of all 124 cases cervical lesion specimens were reviewed and the most representative areas were selected for TMA construction. The TMAs were constructed using a tissue-arraying instrument (Beecher Instruments, Silver Spring, MD, USA), as described in our previous study (17). Briefly, two cores (diameter, 1.5 mm) were removed from the selected area of each donor FFPE specimen and precisely arrayed in a recipient paraffin block. Next, 4-μm thick sections were consecutively incised from the recipient block and transferred to poly-L-lysine-coated glass slides. Hematoxylin and eosin staining was performed on TMA for the confirmation of tumor samples.

### QD IHC

The expression of Beclin-1 and LC3B protein was detected by QD-IHC, using primary rabbit anti-human Beclin-1 polyclonal antibody (1:50; Santa Cruz Biotechnology, Inc., Santa Cruz, CA, USA) and murine anti-human LC3B monoclonal antibody (1:150; Cell Signaling Technology, Inc., Danvers, MA, USA). QD-IHC was performed according to the manufacturer’s instructions (Wuhan Jiayuan Quantum Dot Co., Ltd., Wuhan, China), as described in our previous study (18). The TMAs were observed using an Olympus BX53 fluorescence microscope (Olympus Corporation, Tokyo, Japan) equipped with an Olympus CCD DP73. A positive signal in the cytoplasm or cell membrane is bright red, target-specific and photostable, and the background autofluorescence is green. Negative controls of Beclin-1 and LC3B were carried out by replacing the primary antibodies with Tris-buffered saline.

All immunostained sections were evaluated by two pathologists (Yang GF and Chen HL) with no prior knowledge of the patients’ clinical statuses. Expression was semiquantitatively scored by assessing the intensity of the staining and the percentage of stained cells. The sections were scanned using a high-power field, for calculating the percentage of Beclin-1- and LC3B-positive areas (PAs). PAs were graded as follows: 0, PA ≤20%; 1, PA 20–40%; 2, PA 40–60%; and 3, PA >60%. Subsequently, the intensity of staining (IS) was evaluated in high-power hot-spots and scored as: 0, negative; 1, weak; and 2, strong. Beclin-1 and LC3-B intensity distribution (ID) scores for each case were calculated by the following equation: ID = PA × IS. If the ID was >2, the samples were considered positive; while if the ID was <2, the samples were considered negative, based on the findings of previous reports (19).

### QD FISH

Breifly, 4-μm thick sections of cervical TMAs were deparaffinized, and hydrated sections were hybridized using biotin-labeled HPV 16/18 DNA probes (PanPath B.V., Budel, the Netherlands) and a QD-FISH detection kit (Wuhan Jiayuan Quantum Dot Co., Ltd.). Detection and staining was performed according to the manufacturer’s instructions, and other reagents and steps were the same as those in our previous study involving QD-FISH imaging of Epstein-Barr virus in gastric cancer (11). Red punctate or diffuse nuclear staining was regarded as being positive for HPV16/18 and the background autofluorescence was green. Assessment of HPV16/18 QD-FISH signals was carried out using the Olympus BX53 microscope (CCD DP73; Olympus Corporation). A tissue block from a confirmed HPV-positive cervical carcinoma was used as positive control.

### Statistical analysis

All data were analyzed using SPSS, version 17.0 (SPSS, Inc., Chicago, IL, USA). χ^2^ and Fisher’s exact tests were used to compare different rates of expression. Correlations were calculated using the Spearman’s rank correlation test. A two-tailed P<0.05 was considered to indicate a statistically significant difference.

## Results

### Expression of Beclin-1 and LC3B in normal and cervical cancer tissues

Fluorescent semiconductor nanocrystal QDs are an important class of fluorescent labels used for biological and biomedical imaging (20). In our previous studies, the QD-IHC technique achieved levels of sensitivity and specificity that were sufficient for detecting known expression signals in biopsy and FFPE specimens, enabling high-throughput application (18,19).

**Figure 1:**
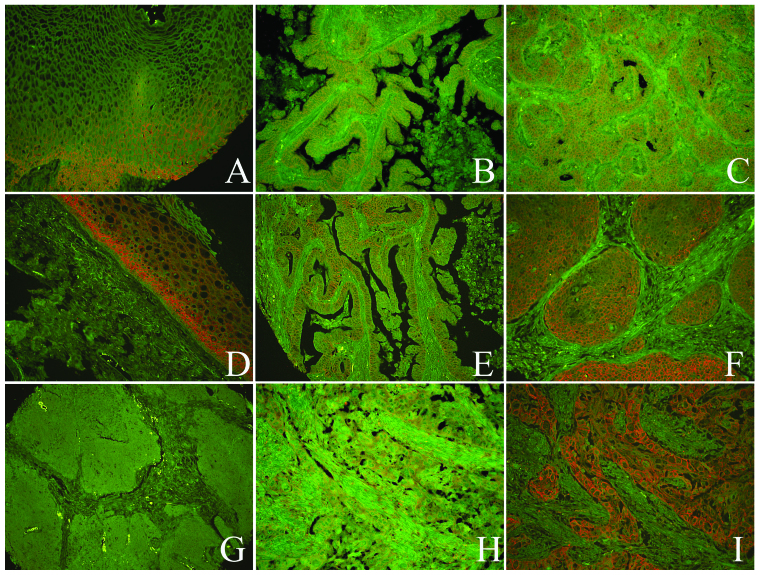
Expression of Beclin-1 and LC3B proteins in the normal cervical and cancerous tissues was analyzed by quantum dot immunofluorescence histochemistry. A positive signal for Beclin-1 was located (A) at the cytoplasm membrane of normal cervical squamous epithelial cells, (B) in the cervical adenocarcinoma cells and (C) in the cervical squamous cell cancer cells. A positive signal for LC3B was observed in the (D) normal cervical squamous epithelial cells, (E) cervical adenocarcinoma cells and (F) cervical squamous cell cancer cells; however, a negative signal for LC3B was detected in the stroma. (G) Beclin-1 and LC3B protein expression was negative in the same cervical SCC case. The expression of (H) Beclin-1 and (I) LC3B proteins were positive at the same cervical SCC case (A–F, H and I, magnification, ×200; G, magnification, ×100). LC3B, microtubule-associated proteins 1A/1B light chain 3B; SCC, squamous cell carcinoma.

In the present study, the subcellular localization and expression of Beclin-1 and LC3B proteins in normal cervical squamous epithelial and cancer tissues was detected via QD-IHC. Specific Beclin-1 staining was predominantly observed at the cytoplasm membrane in normal cervical epithelial and cancer tissues (Fig. 1A–C). In normal cervical squamous epithelial tissues, the positive rate of Beclin-1 of the 20 samples was 90%. By contrast, Beclin-1 was detected in 44% (35/80) of SCCs and 42% (10/24) of adenocarcinomas. The expression of Beclin-1 was significantly decreased in cancer tissues when compared with that in normal cervical tissues (Table II). LC3B was located at the cell membrane and cytoplasm in normal cervical squamous epithelial and cancer tissues (Fig. 1D–F), and no stone-like expression pattern was identified as previously described in breast carcinoma and lung cancer (20,21). In normal cervical tissues, the positive rate of LC3B in the 20 samples was 80%. However, LC3B was detected in 32 (40%) of the 80 SCCs, and 46% (11/24) of adenocarcinomas. A significant decrease in LC3B expression was identified in cancer tissues when compared with that in normal cervical tissues (Table II).

### Clinicopathological significance of Beclin-1 and LC3B in cervical SCC

To investigate the effect of Beclin-1 and LC3B protein expression on malignant progression, the correlation between Beclin-1 and LC3B expression and the clinicopathological features, including age and differentiation grade, were respectively examined. However, as shown in Table I, no significant association was identified between Beclin-1, LC3B and the various clinicopathological features.

### Correlation between Beclin-1 and LC3B in cervical SCCs

The association between Beclin-1 and LC3B (Table I) was further investigated. In 45 cases of Beclin-1-negative expression, 33 (73.3%) cases exhibited negative expression of LC3B (Fig. 1G), and in 35 cases of Beclin-1-positive expression, 20 (57.1%) cases were LC3B-positive (Fig. 1H and I), whereby a positive correlation was identified (r=0.309; P=0.005).

### Clinicopathological significance of negative Beclin-1 and LC3B expression in the cervical SCCs with hrHPV infection

**Figure 2:**
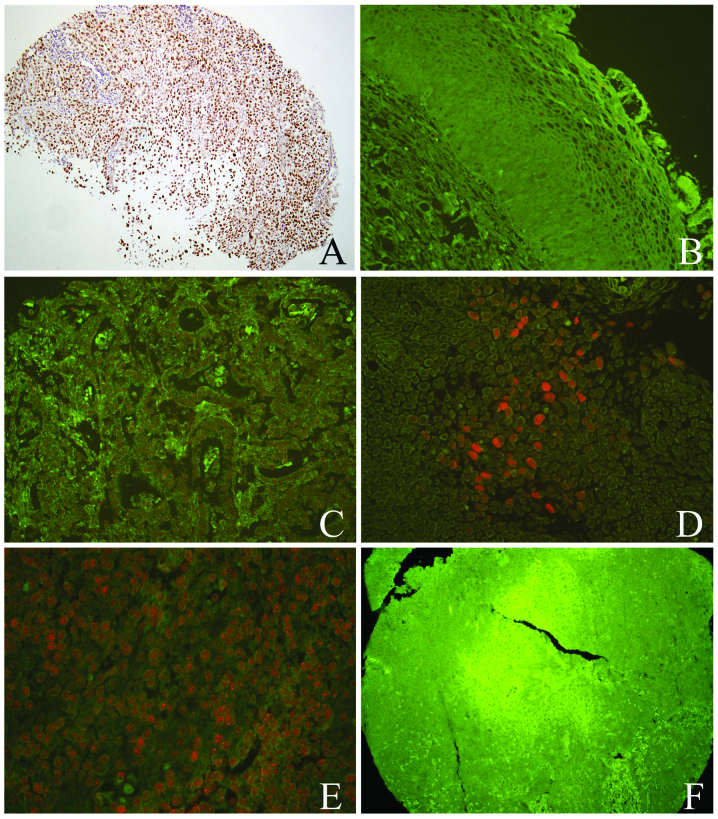
hrHPV infection was detected by quantum dot fluorescence *in situ* hybridization in the normal cervical and cancerous tissues. (A) Positive controls revealed hrHPV located in the nuclei, which was detected by chromogenic in situ hybridization. hrHPV exhibited a positive signal in the (B) normal cervical tissues, (C) adenocarcinoma and (D) SCC. (E and F) In the same SCC case, hrHPV infection exhibited a positive signal (E); however, Beclin-1 exhibited a negative signal (F). (A, B and F, magnification, ×100; C–E, magnification, ×200). hrHPV, high risk human papillomavirus; SCC, squamous cell carcinoma.

The hrHPV infection signal was detected using QD-FISH with biotin-labeled DNA probes. In cervical cancer, the positive hrHPV signal was located in the nuclei of tumor cells (Fig. 2); however, the majority of normal cervical tissues exhibited a negative signal. Positive rates of hrHPV were 83.75% (67/80) for cervical SCC and 62.5% (15/24) for cervical adenocarcinoma, and a significant difference was identified when compared with normal cervical tissues (Table II). In addition, the result of hrHPV infection was evaluated, and Beclin-1 and LC3B were found to negatively correlate with hrHPV infection (Fig. 2E and F; Table II). Simultaneously, the clinicopathological significance of negative Beclin-1 and LC3B expression in cervical SCC with hrHPV infection was investigated. As shown in Table III, the negative expression of Beclin-1 and LC3B in cervical SCC with hrHPV infection was found to promote a higher clinical tumor node metastasis (TNM) stage and lymph node metastasis.

## Discussion

The current study revealed that the expression levels of Beclin-1 and LC3B are significantly lower in cervical cancer tissues than in normal cervical squamous epithelial tissues, and were found to negatively correlate with hrHPV infection. In addition, a positive correlation was identified between Beclin-1 and LC3B protein expression. The results also revealed that the absence of autophagy in combination with hrHPV infection may accelerate cervical SCC progression. Therefore, reduced expression of Beclin-1 and LC3B may be associated with cervical carcinogenesis. Furthermore, the hrHPV-host cell interaction may inhibit autophagy, which may aid virus duplication and infection, and cervical cancer development.

Autophagy is a self-degradation mechanism, which is associated with tumor progression. Recently, the role of autophagy in cancer development and treatment has been investigated *in vitro* and *in vivo* (23). Previous studies have indicated that autophagy is involved in tumor suppressor pathways. Inactivation of autophagy-specific genes, including Beclin-1, results in increased tumorigenesis in mice, and enforcement of the expression of such genes inhibits the formation of human breast tumors in mouse models (24). In the present study, the expression of Beclin-1 and LC3B proteins was significantly decreased in cancer tissues when compared with that of normal cervical tissues, and a positive association was identified between Beclin-1 and LC3B protein expression in cervical SCC, which demonstrated that reduction of autophagy aids cervical carcinogenesis. mRNA and protein levels of Beclin-1 and LC3B are also significantly decreased in lung cancer tissues (25). The positive expression of Beclin-1 in non-Hodgkin lymphomas correlates with the presence of LC3-positive autophagic vacuoles (26). In melanocytic neoplasms, similar results have also been observed, whereby Beclin-1 cytoplasmic protein and mRNA, as well as LC3 mRNA and LC3B protein, significantly decreased with tumor progression (13). However, these results differ from those reported in gastric cancer cells, which revealed that Beclin-1 protein and mRNA expression was significantly higher than that of the corresponding normal tissues (27). In various gastrointestinal cancers, LC3B expression has been found to positively correlate with Ki-67 index in early cancers, indicating that LC3B expression is advantageous for cancer development in the early phases of carcinogenesis (23); however, the mechanism remains unclear.

The association between Beclin-1 and LC3B expression and the clinical characteristics of cervical SCC patients was also analyzed. No significant correlation was identified between the expression of Beclin-1 and LC3B and age, pathological grade, tumor depth, lymph node metastasis, or TNM stage of patients with cervical SCC. Although Beclin-1 and LC3 were not found to be associated with age, FIGO stage, pathological differentiation or lymph node metastasis, they may exhibit prognostic significance in early stage cervical SCC (28). Similar results from previous studies have revealed that the expression of Beclin-1 and LC3B in lung cancer tissues was not affected by patient age, gender, smoking, histological type, lymph node metastasis or TNM stage (25). Furthermore, in esophageal carcinomas, LC3 expression was not found to correlate with various clinicopathological factors, including survival (23). By contrast, significant correlations have been identified between the peripheral intensity level of LC3 expression and tumor size and tumor necrosis of pancreatic cancer (29). These observations highlight the different roles of Beclin-1 and LC3B in the progression of various cancers. In order to utilize the modulation of autophagy for cancer therapy, further investigation with regards to the role of autophagy in human cancers is required.

Cell autophagic machinery is known to capture and degrade intracellular various pathogens, which is an important component of the host response against viral infections (30). Therefore, numerous viruses have developed methods to block autophagy or subvert this mechanism (31). HPV infection induces an autophagic response, including upregulation of marker proteins for autophagy, in host keratinocytes (32). In HeLa cells infected by HPV16 pseudovirions *in vitro*, autophagy was observed to be induced during the HPV16 entry process, which implies that autophagosomes are generated from the plasma membrane as a result of HPV infection (33). The specificity of genetic knockdowns of mTOR, Beclin-1 and Atg7 have confirmed that HPV-induced restraint of autophagy is important for early infection events (31,34).

In the present study, the hrHPV infection signal was detected by QD-FISH using biotin-labeled DNA probes. The positive signal for hrHPV was 83.75% (67/80) in cervical SCC tissues and 62.5% (15/24) in cervical adenocarcinoma, which is significantly higher than that of normal cervical squamous epithelial cells, indicating that hrHPV infection is involved in the development of cervical cancer (35). In addition, the result of hrHPV infection was investigated, and significant negative correlations were detected between Beclin-1, LC3B and hrHPV infection, indicating that hrHPV infection may inhibit autophagy. It is hypothesized that the HPV-host cell interaction stimulates the PI3K/Akt/mTOR pathway and inhibits autophagy, and in combination these events aid virus infection (36). In addition, persistent HPV infection may stabilize the ATPase family AAA domain containing 3A (an anti-autophagy factor) expression to inhibit cell autophagy and apoptosis, as well as increasing drug resistance in uterine cervical cancer (37). Simultaneously, the clinicopathological significance of negative Beclin-1 and LC3B expression in cervical SCC with hrHPV infection was investigated, and the results revealed that negative expression of Beclin-1 and LC3B in cervical SCC with hrHPV infection promoted a higher clinical TNM stage and lymph node metastasis, indicating that the absence of autophagy combined with hrHPV infection may accelerate cervical SCC progression. In addition, the mTOR pathway is important in cervical carcinogenesis and thus, targeted therapies may be developed for SCC as well as its precursor lesion, high-grade squamous intraepithelial lesion (38).

In conclusion, this study revealed that the decreased expression of Beclin-1 and LC3B may be involved in cervical carcinogenesis. The absence of autophagy combined with hrHPV infection may accelerate cervical SCC progression. The results highlight the clinical importance of the hrHPV-host cell interaction, which may inhibit autophagy, aiding virus duplication and infection, as well as cervical cancer development. However, further studies are required to clarify the mechanism with regards to hrHPV regulation of the autophagy signaling pathway and its involvement in the development and progression of cervical cancer.

## Figures and Tables

**Table I:** Correlation between Beclin-1 and LC3B protein expression and clinicopathological parameters of cervical squamous cell carcinomas.

		Beclin-1, n (%)			LC3B, n (%)		
					
Group	Cases, n	−	+	P-value	−	+	P-value
Age				0.463			0.715
≥45	38	23 (61)	15 (39)		22 (58)	16 (42)	
<45	42	22 (52)	20 (48)		26 (62)	16 (38)	
Grade				0.723			0.180
I + II	28	15 (54)	13 (46)		14 (50)	14 (50)	
III	52	30 (58)	22 (42)		34 (65)	18 (35)	
Tumor stage				0.159			0.079
T1	65	39 (60)	26 (40)		42 (65)	23 (35)	
T2 + T3	15	6 (40)	9 (60)		6 (40)	9 (60)	
TNM stage				0.219			0.232
I + II	56	29 (52)	27 (48)		36 (64)	20 (36)	
III + IV	24	16 (67)	8 (33)		12 (50)	12 (50)	
Lymph node status				0.127			0.364
N0	57	29 (51)	28 (49)		36 (63)	21 (37)	
N1–3	23	16 (70)	7 (30)		12 (52)	11(48)	
hrHPV infection				0.008			0.019
Negative	13	3 (23)	10 (77)	r = −0.295	4 (30)	9 (70)	r = −0.263
Positive	67	42 (63)	25 (37)		44 (66)	23 (34)	
Beclin-1				-			0.005
Negative	45	-	-		33 (73)	12 (27)	r = 0.309
Positive	35	-	-		15 (43)	20 (57)	

LC3B, light chain 3B; TNM, tumor node metastasis; hrHPV, high risk human papillomavirus.

**Table II:** Expression of Beclin-1 and LC3B proteins, and hrHPV infection in the different cervical lesion tissues.

		Beclin-1, n(%)			LC3B, n(%)			hrHPV, n(%)		
							
Different groups	Cases, n	−	+	P-value	−	+	P-value	−	+	P-value
Normal cervical epithilium	20	2 (10)	18 (90)	0.000^a^	4 (20)	16 (80)	0.001^a^	18 (90)	2 (10)	0.001^a^
SCC	80	45 (56)	35 (44)	0.001^b^	48 (60)	32 (40)	0.020^b^	13 (16)	67 (84)	0.001^b^
Adenocarcinoma	24	14 (58)	10 (42)	0.857^c^	13 (54)	11 (46)	0.611^c^	9 (37)	15 (63)	0.025^c^

LC3B, light chain 3B; hrHPV, high risk human papillomavirus; SCC, squamous cell carcinoma.

a Normal cervical epithelium vs. SCC;

b normal cervical epithelium vs. adenocarcinoma;

c SCC vs. adenocarcinoma.

**Table III:** Clinicopathological significance of negative Beclin-1 and LC3B expression in cervical squamous cell carcinomas with hrHPV infection.

		Beclin-1, n(%)			LC3B, n(%)		
					
Group	Cases, n	−	+	P-value	−	+	P-value
Grade				0.451			0.254
I + II	23	13 (57)	10 (43)		13 (57)	10 (43)	
III	44	29 (66)	15 (34)		31 (70)	13 (30)	
Tumor stage				0.482			0.293
T1	57	37 (65)	20 (35)		39 (68)	18 (32)	
T2 + T3	10	5 (50)	5 (50)		5 (50)	5 (50)	
TNM stage				0.083			0.044
I + II	48	27 (56)	21 (44)		28 (58)	20 (42)	
III + IV	19	15 (79)	4 (21)		16 (84)	3 (16)	
LN status				0.034			0.065
N0	49	27 (55)	22 (45)		29 (59)	20 (41)	
N1–3	18	15 (83)	3 (17)		15 (83)	3 (17)	

LC3B, light chain 3B; hrHPV, high risk human papillomavirus; TNM, tumor node metastasis; LN, lymph node.
